# 

**Published:** 1861-07

**Authors:** 


### CONTENTS.

| ART. | | PAGE |
|---|---|---|
| | Quarterly Retrospect | xxxi—xlvi |
| I. | The Vacation | 349 |
| II. | On the Distinction between Analytical and Synthetical Judgments. By C. M. Ingleby, LL.D. | 357 |
| III. | The Great Arcanum:—The Turkish Bath | 366 |
| IV. | The Amenities of Statistics | 378 |
| V. | Who is a Doctor? | 394 |
| VI. | The Gheel Question. By J. Munday, M.D., of Moravia | 399 |
| VII. | Three Thousand a Year:—A Soliloquy | 412 |
| VIII. | The State of Lunacy in Scotland | 416 |
| IX. | The Road Murder Psychologically considered | 424 |
| X. | Miscellanea Medica | 444 |
| XI. | Cottage Asylums:—A Sequel. By W. A. F. Browne, One of the Commissioners in Lunacy for Scotland | 449 |
| XII. | Medical Gossip | 478 |
| XIII. | Literary Gossip and Record | 486 |
| | Foreign Medico-Psychological Literature | 507 |
| | The Medical Profession of Scotland, and the Lunacy Act of that Kingdom | 519 |

No. IV. will be published on the 1st of October.

